# Combined Pulmonary Fibrosis and Emphysema: Current Status and Future Perspective

**DOI:** 10.1111/crj.70236

**Published:** 2026-11-03

**Authors:** Qinyu Deng, Chen Chen, Qinghe Zhang, Zilin Wei, Jianqi Sun, Feng Li

**Affiliations:** ^1^ Department of Basic Medical Sciences, School of Medicine Shanghai Jiao Tong University Shanghai People's Republic of China; ^2^ School of Biomedical Engineering Shanghai Jiao Tong University Shanghai People's Republic of China; ^3^ Department of Pulmonary and Critical Care Medicine, Shanghai Chest Hospital, School of Medicine Shanghai Jiao Tong University Shanghai People's Republic of China

**Keywords:** artificial intelligence, combined pulmonary fibrosis and emphysema, etiologic classification, risk stratification

## Abstract

**Background:**

Combined pulmonary fibrosis and emphysema (CPFE) is a clinical syndrome defined by the coexistence of upper‐lobe emphysema and lower‐lobe pulmonary fibrosis. It is characterized by clinical, functional, and radiologic features that differ significantly from isolated idiopathic pulmonary fibrosis or chronic obstructive pulmonary disease.

**Summary:**

This review summarizes recent advances in CPFE, focusing on etiologic classification, clinical and radiologic features, diagnosis and treatment, prognostic evaluation, and emerging perspectives in artificial intelligence (AI). Based on current research, we summarize the etiologic classification of CPFE into four types: smoking‐related, systemic autoimmune rheumatic disease‐associated, occupational‐ and environmental‐related, and idiopathic. These types differ in epidemiologic characteristics, imaging patterns, and disease progression. Consequently, future research is expected to focus on mechanism‐driven phenotyping and quantitative imaging analysis, where AI may provide additional value in disease identification, risk stratification, and dynamic prognostic assessment.

**Key Message:**

This review proposes a novel framework for CPFE, integrating etiologic subtyping and a diagnostic algorithm to guide precise management, and highlights AI‐powered strategies for future personalized interventions.

## Graphical abstract

This review summarizes recent advances in combined pulmonary fibrosis and emphysema (CPFE), proposing a novel etiologic classification framework encompassing four subtypes. We integrate a diagnostic algorithm to guide precise management and highlight emerging artificial intelligence strategies for risk stratification and dynamic prognostic assessment. These insights pave the way for mechanism‐driven phenotyping and personalized interventions.

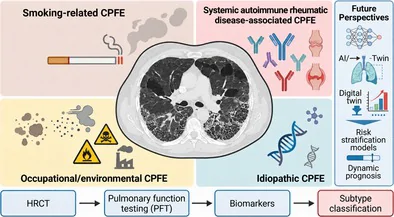

## 1 Introduction

Combined pulmonary fibrosis and emphysema (CPFE), first systematically described by Cottin et al. [1], is a syndrome characterized by the coexistence of emphysema predominantly affecting the upper lobes and pulmonary fibrosis mostly affecting the lower lobes. Emphysema is common in interstitial lung disease (ILD) patients who are current or former smokers, and CPFE accounts for 26%–54% of patients with idiopathic interstitial pneumonia (IIP). The prevalence of CPFE in patients with chronic obstructive pulmonary disease (COPD) is 5.6% [2, 3]. The prevalence of CPFE in the general population remains unclear. Chae et al. reported a CPFE prevalence of 3.1% in a cohort of 2016 asymptomatic Korean male smokers aged ≥ 40 years [4].

An international research statement on CPFE indicated that CPFE is not merely a simple combination of two diseases, but an independent entity with unique pathophysiological mechanisms, natural course, and comorbidities [3]. CPFE itself exhibits significant heterogeneity in etiology and radiologic distribution. Based on recent advances in research, this review summarizes the potential etiology of CPFE, refines the diagnostic and differential diagnosis process, elucidates the severity assessment, prognosis evaluation, and treatment strategy, and proposes future perspectives on artificial intelligence (AI) applications in the diagnosis and management of CPFE.

## 2 Main Text

### 2.1 Etiology of CPFE

CPFE is not attributable to a single etiology, but rather represents a heterogeneous clinical syndrome characterized by overlapping radiologic and physiologic manifestations arising from diverse underlying mechanisms. Based on current evidence, the major factors implicated in CPFE development can be broadly classified into four categories (shown in Figure 1). However, these categories should not be regarded as mutually exclusive, as multiple contributing factors may coexist in individual patients.

**FIGURE 1:**
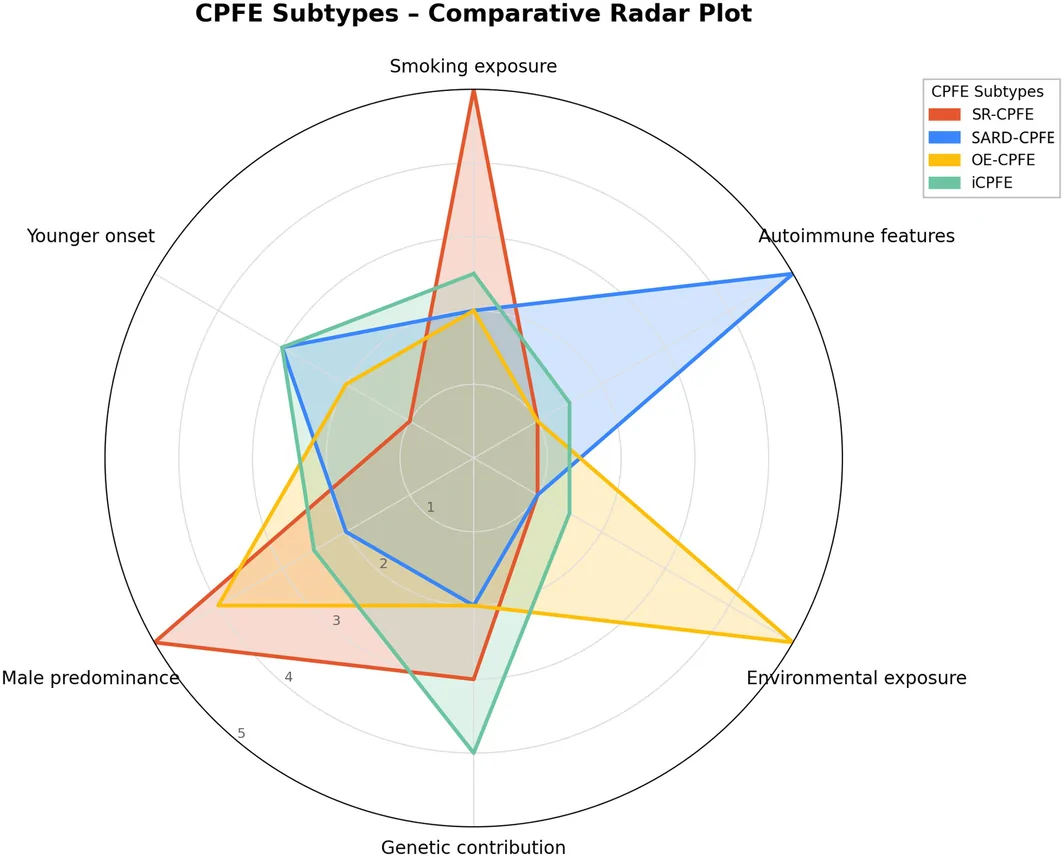
Comparative radar plot of CPFE subtypes. This radar plot summarizes the characteristic risk profiles of the four major CPFE subtypes across six dimensions, illustrating how smoking, autoimmunity, environmental exposures, genetic susceptibility, sex distribution, and age of onset differentially shape each phenotype. iCPFE = idiopathic CPFE; OE‐CPFE = occupational and environmental‐related CPFE; SARD‐CPFE = systemic autoimmune rheumatic disease‐associated CPFE; SR‐CPFE = smoking‐related CPFE.

#### 2.1.1 Smoking‐Related CPFE (SR‐CPFE)

Smoking is a well‐established risk factor for CPFE. Smokers have approximately twice the risk of developing CPFE compared with nonsmokers [5], and about 70%–90% of affected patients report a long history of smoking, often exceeding 40 pack‐years. Cohort studies further demonstrate a clear dose–response relationship between smoking intensity and pulmonary fibrosis severity [6]. Most patients with SR‐CPFE are male [7]. SR‐CPFE represents the most extensively studied phenotype within the CPFE spectrum. At the molecular level, smoking‐related metabolites can directly contribute to the development of CPFE. Nicotine metabolites can induce abnormal hyaluronan accumulation via the adenosine A2B receptor‐hyaluronan synthase 2 (ADORA2B‐HAS2) pathway and activate the hyaluronic acid‐CD44 (HA‐CD44) signaling cascade. Both processes promote fibroblast activation and disrupt alveolar architecture, thereby promoting fibrotic remodeling characteristic of CPFE [8]. Nevertheless, smoking exposure alone does not necessarily define SR‐CPFE, as tobacco exposure may lead to other respiratory diseases or pathological processes, such as idiopathic pulmonary fibrosis (IPF) [9]. In this review, SR‐CPFE is defined as CPFE cases in which smoking is considered the predominant identifiable contributing factor based on comprehensive clinical evaluation.

#### 2.1.2 Systemic Autoimmune Rheumatic Disease‐Associated CPFE (SARD‐CPFE)

Recent studies have highlighted an autoimmune hypothesis in the pathogenesis of CPFE. Tzouvelekis et al. reported that 42.5% of CPFE patients were positive for antinuclear antibodies (ANA), and 17.5% were positive for myeloperoxidase‐antineutrophil cytoplasmic antibody (MPO‐ANCA). CD20^+^ B‐cell lymphoid follicles were also identified in fibrotic regions, and their density positively correlated with patient survival. These findings suggest that immune dysregulation may contribute to a distinct CPFE phenotype [10]. SARD‐CPFE represents a clinically recognizable phenotype in which an established SARD is considered the predominant contributing factor. It has been reported in patients with various SARDs, including systemic sclerosis (SSc), rheumatoid arthritis (RA), polymyositis/dermatomyositis (PM/DM), and ANCA‐associated vasculitis (AAV) [11, 12]. Compared with SR‐CPFE, SARD‐CPFE is more frequently observed in females or individuals with minimal or no smoking exposure. However, a cohort study of 976 patients with RA showed that, even in the context of SARD, male sex and a history of current or former smoking were still associated with a higher risk of developing CPFE [13]. These findings suggest that both immune‐related processes and smoking‐related pathogenic mechanisms may interact with each other and contribute to CPFE development. Commonly positive markers include anti‐Scl‐70, anti‐PL‐7, anti‐PL‐12, anti‐Jo‐1, other antisynthetase antibodies, and ANCA, particularly MPO‐ANCA [14]. These specific autoantibodies help identify the underlying type of SARD, enabling risk stratification and guiding more personalized treatment strategies.

#### 2.1.3 Occupational and Environmental‐Related CPFE (OE‐CPFE)

OE‐CPFE, an important yet often underrecognized cause of CPFE, is associated with the inhalation of specific inorganic or organic dusts or chemicals, or exposure to certain medications. Existing studies have demonstrated that various occupational and environmental exposures, including coal dust, asbestos, silica, and pesticides, are associated with the development and progression of CPFE [3]. OE‐CPFE can be viewed as a distinct pulmonary phenotype due to the interaction between environmental exposures and the host immune response. In clinical practice, meticulous assessment of exposure history is essential for facilitating diagnosis.

#### 2.1.4 Idiopathic CPFE (iCPFE)

iCPFE represents a clinical phenotype of CPFE in which no predominant identifiable underlying cause is established after comprehensive clinical evaluation. Emerging evidence suggests that genetic susceptibility, particularly alterations in telomere biology, may increase vulnerability to pulmonary fibrosis [15]. For example, pathogenic variants in telomere‐related genes such as telomerase reverse transcriptase (TERT), telomerase RNA component (TERC), nuclear assembly factor 1 (NAF1), and desmoplakin (DSP) [15, 16, 17] have been associated with pulmonary fibrosis and may contribute to CPFE susceptibility. In addition, alterations in surfactant homeostasis may represent another potential genetic pathway. Pathogenic variants in surfactant‐related genes such as ATP Binding Cassette Subfamily A Member 3 (ABCA3) and Surfactant Protein C (SFTPC) [18] are well‐established causes of childhood ILD, often associated with severe early‐onset disease. However, recent evidence indicates that some individuals carrying these variants may also develop ILD in adulthood [19], suggesting a broader age‐dependent clinical spectrum. Furthermore, polymorphisms in TGF‐β1‐related pathways may predispose patients with pulmonary fibrosis to emphysematous changes and contribute to CPFE development [20]. Other genetic variants, such as the Gly82Ser variant in the receptor for advanced glycation end products (RAGE) gene, may also contribute to CPFE susceptibility by impairing interactions between lung interstitial cells and the extracellular matrix [21]. Collectively, these findings suggest that specific genetic backgrounds may reduce the lung's tolerance to injury from other causes, thereby predisposing susceptible individuals to develop CPFE [22].

### 2.2 Features of CPFE

#### 2.2.1 Symptoms and Signs

CPFE patients exhibit heterogeneous clinical manifestations. Common symptoms include exertional dyspnea, cough, sputum production, chest pain, and wheezing [1, 23, 24]. Common physical findings include Velcro crackles and clubbing [1]. Most patients are in New York Heart Association (NYHA) functional Class III or IV [1, 3, 25]. Patients in later stages may show hypoxemia on minimal exertion or exercise intolerance [24].

#### 2.2.2 Imaging Features

Emphysema typically manifests as centrilobular, panlobular, or paraseptal patterns and predominantly involves the upper lobes [3]. Paraseptal emphysema is particularly common and considered a characteristic feature of CPFE [1, 3]. Fibrosis typically manifests as reticular opacities, honeycombing, ground‐glass opacities, and traction bronchiectasis and predominantly involves the lower lobes. Usual interstitial pneumonia (UIP) is the most common fibrotic pattern, whereas alveolar macrophage pneumonia (AMP), nonspecific interstitial pneumonia (NSIP), airspace enlargement with fibrosis (AEF), and other patterns may also be observed [1] (shown in Figure 2). Brillet et al. described three distinct distribution patterns of emphysema and fibrosis in CPFE: diffuse emphysema (centrilobular and/or panlobular) with a transition zone between bullae and honeycombing, paraseptal emphysema predominantly featuring enlarged basilar subpleural bullae, and separate processes with discrete areas of fibrosis and emphysema [26]. Distribution patterns may reflect disease status. For example, the combined pattern may indicate a more active disease process, whereas the separate pattern may suggest a relatively stable state. Kinoshita et al. demonstrated the coexistence of emphysema and fibrosis in each lung lobe using autopsy and surgical lung resection specimens from patients with IPF and emphysema [27]. A key feature of CPFE is the presence of AEF or thick‐walled large cysts (TWLCs), which are often > 2 cm in diameter with a wall thickness > 1 mm. TWLCs differ from simple honeycombing, where cysts are smaller and have thinner walls [28]. TWLCs may result from fibrosis‐induced traction distension and wall thickening of emphysematous areas, reflecting the interaction between emphysema and fibrosis.

**FIGURE 2:**
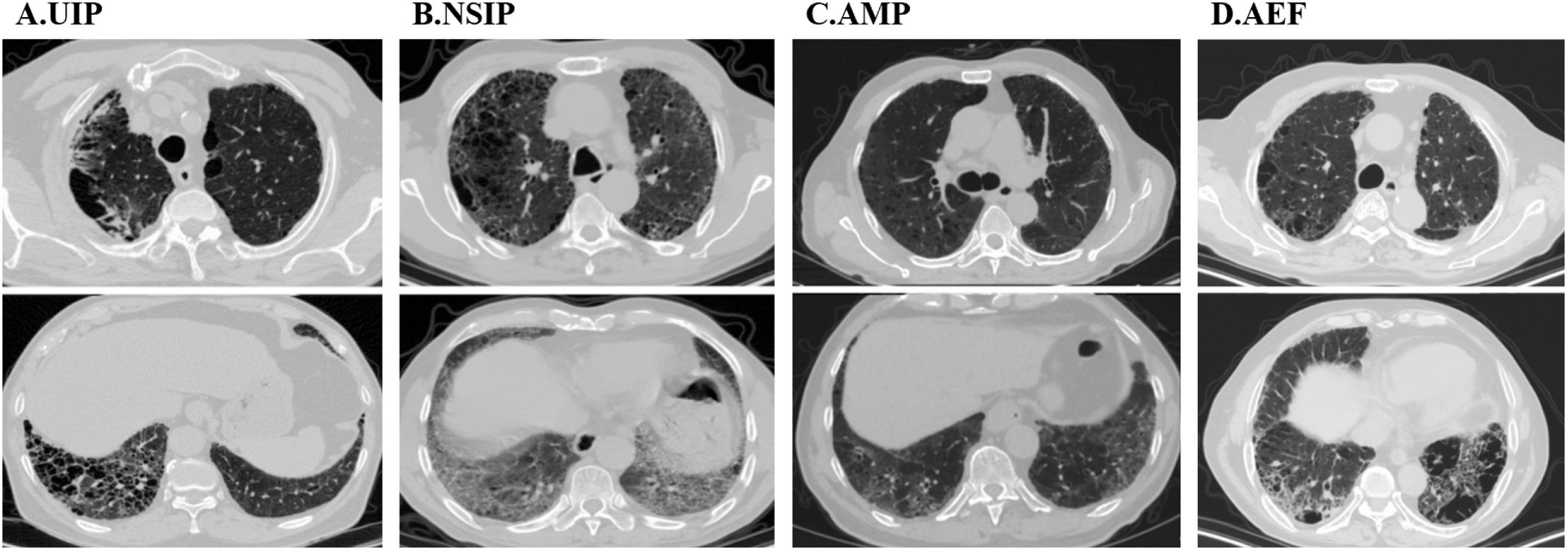
High‐resolution CT (HRCT) manifestations of the emphysematous and fibrotic regions of the lungs in patients with different types of CPFE. (a) Usual interstitial pneumonia (UIP); (b) nonspecific interstitial pneumonia (NSIP); (c) alveolar macrophage pneumonia (AMP); (d) airspace enlargement with fibrosis (AEF).

#### 2.2.3 Pulmonary Function

Due to the counterbalancing effects of increased lung compliance from emphysema and decreased compliance from pulmonary fibrosis, lung volume and flow parameters are often relatively preserved compared to patients with IPF alone, such as total lung capacity (TLC), forced vital capacity (FVC), forced expiratory volume in 1 s (FEV_1_), and the FEV_1_/FVC ratio [3]. This counterbalancing effect often results in seemingly “normal” lung volumes and ventilatory function in CPFE patients, thereby masking severe parenchymal destruction. In contrast to this “pseudonormal” presentation, gas exchange is significantly impaired, as manifested by a remarkable reduction in the diffusing capacity of the lungs for carbon monoxide (DLCO), which represents the most characteristic pulmonary functional abnormality of CPFE [29]. The mechanisms involve the combined effects of alveolar wall destruction, interstitial thickening, and other factors.

### 2.3 Diagnosis of CPFE

#### 2.3.1 Initial Assessment of Symptoms, Medical History, and Physical Examination

The diagnostic process for CPFE begins with the patient's chief complaints and a detailed medical history. Chronic and progressive dyspnea, particularly exertional shortness of breath accompanied by reduced exercise tolerance, is the most common initial presentation. Patients may also seek medical attention because of exertional hypoxemia or recurrent lower respiratory tract infections. Key elements of the history include sex, smoking status, occupational or environmental exposures, use of medications, family history of lung disease, and history of SARD. These factors influence the etiologic assessment and differential diagnosis of CPFE [3]. Physical examination is mandatory. Clubbing, bibasilar crackles, and scattered rhonchi may be present but are often nonspecific.

#### 2.3.2 Imaging Screening

The imaging features of CPFE are highly characteristic and diagnostically informative. Accordingly, HRCT is essential, as it identifies the hallmark coexistence of emphysema and pulmonary fibrosis, distinguishes CPFE from other lung conditions, and guides further invasive procedures or quantitative/qualitative analyses [30]. Regarding the threshold for the extent of emphysema on CT, our research group tends to use 10% because it aligns with Global Initiative for Chronic Obstructive Lung Disease (GOLD) stage II or higher in individuals with isolated COPD, implying that such an extent of emphysema typically leads to notable symptomatic and physiologic impacts [31]. Furthermore, prior studies have successfully utilized this 10% benchmark to diagnose CPFE, demonstrating excellent inter‐rater agreement (κ = 0.89) [32]. While CPFE exhibits common radiologic features across different etiologies, each subtype may present additional specific imaging findings that aid in etiologic differentiation and diagnosis. SR‐CPFE is primarily characterized by predominant emphysema. In a single‐center retrospective study including 188 CPFE patients [5], smokers had higher visual scores for centrilobular emphysema (CLE) compared with nonsmokers, and emphysema progression during follow‐up was also more frequent. iCPFE is characterized by upper‐lobe emphysema combined with lower‐lobe UIP‐type fibrosis, which serves as a key feature to distinguish it from other CPFE subtypes. In contrast, SARD‐CPFE generally exhibits less extensive fibrosis and emphysema [33], and its ground‐glass opacities often reflect inflammatory activity rather than established fibrosis [3]. There is currently no consensus on whether these inflammatory changes should be included when assessing the severity of fibrosis in CPFE. The diagnosis of OE‐CPFE focuses on recognition of exposure‐specific imaging features. Patients with asbestosis may present with pleural plaques, diffuse pleural thickening, and subpleural lines, whereas honeycombing is uncommon [34]. In silicosis, large nodules with “eggshell” calcified lymph nodes may be observed. These features help differentiate OE‐CPFE from other CPFE subtypes. When HRCT results are inconclusive or inconsistent with the clinical presentation, multidisciplinary discussion is recommended.

#### 2.3.3 Pulmonary Function and Blood Gas Analysis

As previously noted, the functional profile of CPFE is characterized by relatively preserved or mildly reduced lung volumes alongside apparently decreased DLCO. We recommend measuring the annual decline rates of FVC, FEV_1_, FEV_1_/FVC, and DLCO, which can assist in differential diagnosis of CPFE from isolated IPF or COPD [30]. The 6‐min walk test (6MWT) or cardiopulmonary exercise testing (CPET) can evaluate cardiopulmonary function, exercise‐induced hypoxia, and prognostic risk. Additionally, the FEV_1_/DLCO ratio may also serve as an indicator of concomitant pulmonary vascular disease and prompt additional diagnostic evaluation [35]. Differences in arterial oxygen pressure between rest and exercise, along with arterial blood gas analysis data, should also be recorded, which are crucial for severity stratification and follow‐up monitoring.

#### 2.3.4 Screening for Underlying Causes

After confirming the existence of CPFE, underlying causes should be investigated. Given the important role of autoimmune mechanisms in the onset and progression of CPFE, a systematic screening for autoantibodies, such as ANA, extractable nuclear antigen (ENA) antibodies, anti‐Scl‐70, antineutrophil cytoplasmic antibodies (ANCA) including anti‐proteinase 3 antibody (anti‐PR3) and anti‐myeloperoxidase antibody (anti‐MPO), or myositis‐specific antibodies, is recommended [7, 36]. This not only helps identify an underlying SARD [10] but also provides a key molecular basis for subsequent implementation of disease‐specific immunomodulatory therapy [37]. A detailed occupational/environmental exposure history, medication history (such as certain chemotherapeutic agents or antiarrhythmics), family history, and genetic testing when necessary (e.g., in cases of familial fibrosis or early‐onset disease) should also be considered. When the history is unclear, clinical features are ambiguous, or HRCT findings are atypical, bronchoscopy or surgical lung biopsy may be considered following a multidisciplinary evaluation to confirm the histopathological features of CPFE.

#### 2.3.5 Biomarkers

Biomarkers in serum, sputum, or bronchoalveolar lavage (BAL) fluid across different subtypes show potential for aiding diagnosis, assessing fibrosis activity, and predicting prognosis. Serum Krebs von den Lungen‐6 (KL‐6) levels are elevated in patients with CPFE. When distinguishing CPFE from IPF, a KL‐6 cutoff of ≥ 245.4 U/mL yields a sensitivity of 81% and a specificity of 73% [38]. In another study comparing CPFE with COPD [39], the mean KL‐6 level in CPFE was 575 U/mL, higher than 339 U/mL in COPD, and a cutoff of ≥ 551 U/mL can be used for differential diagnosis. Pulmonary surfactant protein A and D (SP‐A and SP‐D) also have potential diagnostic utility in differentiating healthy controls from patients with emphysema, IPF and CPFE [40]. The airway‐derived protein CC16 is elevated in CPFE, reflecting epithelial cell injury [41]. Recent studies further propose that elastin degradation products, such as desmosine and isodesmosine, may serve as potential biomarkers indicating the transition from emphysema to fibrosis [42]. Although these findings provide new insights into CPFE biomarker research, most evidence comes from small cohorts or histology‐based or hypothesis‐driven studies. Therefore, their diagnostic and prognostic value remains to be validated, and these biomarkers cannot replace comprehensive radiologic and physiologic assessment in the evaluation of CPFE (shown in Figure 3).

**FIGURE 3:**
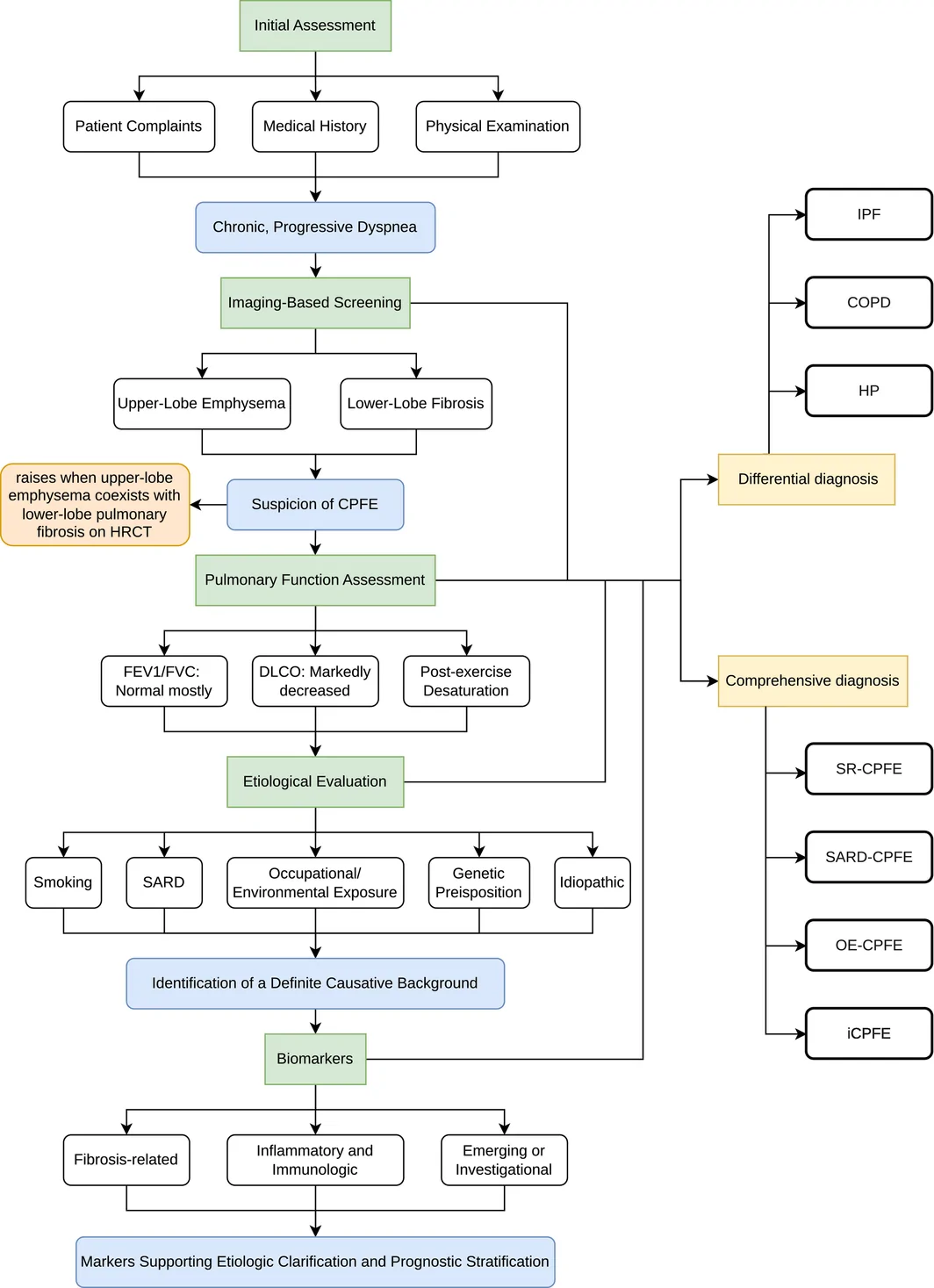
The algorithm of CPFE diagnosis. This algorithm begins with clinical evaluation (symptoms, exposure history, and physical examination) and high‐resolution CT to identify upper‐lobe emphysema coexisting with lower‐lobe fibrosis. Pulmonary function tests showing preserved FEV_1_/FVC but remarkably reduced DLCO support the diagnosis. Subsequent etiologic screening, including smoking, connective tissue disease, occupational exposure, and the absence of an identifiable cause, classifies CPFE into subtypes, whereas biomarkers aid refinement of diagnosis and prognosis.

### 2.4 Differential Diagnosis of CPFE

IPF typically manifests as a UIP pattern dominated by posterior‐basal involvement in the lower lobes, featuring honeycombing and traction bronchiectasis, without significant emphysema. IPF exhibits typical restrictive ventilation with significantly decreased FVC and DLCO and normal FEV_1_/FVC ratio. COPD is primarily characterized by reduced FEV_1_/FVC, increased TLC and residual volume (RV), and sometimes decreased DLCO. Additionally, careful differentiation is required between diffuse lower lobe bullae and the honeycomb pattern in CPFE fibrosis. The former presents as “thin‐walled” hypodense areas. Hypersensitivity pneumonitis (HP) can present with nonfibrotic or fibrotic bronchiolocentric interstitial pneumonia (BIP) patterns on imaging [43]. Fibrotic BIP (in the context of HP) presents with bilateral peripheral bronchovascular interstitial thickening, traction bronchiectasis, and honeycombing, accompanied by ground‐glass opacities and mosaic attenuation (triple density sign) [44]. Fibrosis in fibrotic HP typically presents diffusely or in the upper and middle lung regions, whereas fibrosis in CPFE is predominantly concentrated in the lower lobes. In such patients, detailed occupational/environmental exposure history, specific serological testing, and BAL results are crucial for differentiation (shown in Table 1).

**TABLE 1:** Key features differentiating CPFE from IPF, COPD, and HP.

Differential target	Clinical context (population/symptoms)	Smoking/exposure history	Key HRCT features	Pulmonary function (typical)	Etiologic/contextual clues (useful for differentiation)
CPFE	Typically affects middle‐aged to older adults, predominantly males. Characterized by progressive exertional dyspnea; digital clubbing may be present	Heavy and long‐term smoking is common; occupational exposure may also contribute	Upper‐lobe–predominant emphysema (usually centrilobular or paraseptal) coexisting with lower‐lobe/posterior‐basal fibrosis patterns, such as honeycombing or thick‐walled large cyst	FVC and TLC relatively preserved or mildly reduced; DLCO markedly reduced (often profound); FEV_1_/FVC normal or mildly decreased	Strong smoking history; consider concomitant connective tissue disease markers or definite exposure history
IPF	Middle‐aged to elderly adults with progressive dyspnea and dry cough	Smoking is a risk factor, but not essential	Lower‐lobe and subpleural posterior‐basal–predominant fibrosis with a classic UIP pattern: honeycombing, reticular opacities, and traction bronchiectasis or bronchiolectasis	Restrictive defect (reduced FVC and TLC); DLCO reduced, though CPFE typically shows more profound DLCO impairment due to concomitant emphysema	“Idiopathic” diagnosis based on exclusion of CTD or exposure; radiographic UIP pattern supports consideration of IPF
COPD	Common in smokers: chronic cough, sputum production, and intermittent or progressive dyspnea	Strongly associated with heavy smoking or occupational dust exposure	Predominantly thin‐walled low‐attenuation areas consistent with emphysema; airway wall thickening may be prominent; fibrosis is minimal or absent	Airflow obstruction with reduced FEV_1_/FVC (< 70%); TLC and RV typically increased; DLCO may decrease, but usually less than in CPFE	Chronic cough with sputum and significant airflow obstruction; absence of lower‐lobe–predominant fibrosis or honeycombing on HRCT favors COPD over CPFE
HP	Clear or insidious exposure history; may present with episodic/remitting symptoms or chronic progressive disease	Typically related to repeated inhalation of organic antigens; smoking may modify radiologic appearance	HRCT reveals a BIP pattern characterized by diffuse mosaic attenuation, air‐trapping, and small‐airway disease. Fibrotic BIP may develop in chronic cases; emphysema may be possible in long‐term or smoking‐related presentations	Restrictive or mixed pattern with reduced DLCO; airway‐predominant disease may show partial obstruction	Detailed exposure history, occupational/environmental assessment, antigen‐specific IgG, and BAL lymphocytosis help differentiate HP from CPFE and other ILDs

*Note:* This table summarizes each disorder according to smoking or environmental risk factors, predominant HRCT pattern, and physiologic features, while highlighting the additional diagnostic clues, such as autoantibodies, exposure history, or systemic manifestations, which distinguish CPFE from other conditions.

Abbreviations: BAL = bronchoalveolar lavage; BIP = bronchiolocentric interstitial pneumonia (radiologic/histologic pattern); COPD = chronic obstructive pulmonary disease; CPFE = combined pulmonary fibrosis and emphysema; DLCO = diffusing capacity of the lungs for carbon monoxide; FEV_1_ = forced expiratory volume in 1 s; FVC = forced vital capacity; HP = hypersensitivity pneumonitis (multidisciplinary diagnosis); HRCT = high‐resolution computed tomography; IgG = immunoglobulin G; ILD = interstitial lung disease; IPF = idiopathic pulmonary fibrosis; SARD = systemic autoimmune rheumatic disease; TLC = total lung capacity; UIP = usual interstitial pneumonia.

Beyond distinguishing CPFE from other pulmonary diseases, it is also important to identify the specific underlying SARD in patients with SARD‐CPFE. In particular, patients with MPO‐ANCA positivity should be evaluated for AAV, especially microscopic polyangiitis (MPA), with careful assessment for systemic vasculitic manifestations, including renal involvement, cutaneous vasculitis, and peripheral neuropathy [45]. Importantly, MPO‐ANCA positivity alone does not necessarily indicate established systemic AAV, as ANCA‐positive fibrotic ILD may occur in the absence of overt systemic vasculitic manifestations [37]. Therefore, serologic findings should be interpreted in conjunction with extrapulmonary clinical manifestations to facilitate accurate etiologic attribution and guide subsequent clinical management.

### 2.5 Treatment for CPFE

#### 2.5.1 Smoking Cessation and Avoidance of Environmental Exposures

Given the strong epidemiological and mechanistic association between smoking and CPFE (particularly SR‐CPFE), complete smoking cessation remains a fundamental component of disease management. Therefore, all patients, regardless of disease stage, should receive structured smoking‐cessation interventions, including behavioral counseling and pharmacologic therapy (e.g., varenicline, bupropion, or nicotine replacement [46]).

Strict avoidance of relevant occupational or environmental hazards is equally essential with respect to OE‐CPFE. Exposure reduction may halt further injury to the alveolar–interstitial interface, thereby slowing disease progression. Clinicians should routinely evaluate exposure histories, implement workplace control measures, and recommend personal protective equipment when avoidance is not immediately feasible [47].

#### 2.5.2 Antifibrotic Therapy

Evidence indicates that nintedanib can attenuate the annual decline in FVC in IPF patients with concomitant emphysema. Consequently, nintedanib is considered a potential “disease‐modifying” agent for CPFE in clinical practice [48]. Relevant studies demonstrate that antifibrotic therapies can indeed slow FVC decline in patients with progressive pulmonary fibrosis (PPF). Nerandomilast, a highly selective phosphodiesterase 4B inhibitor capable of inhibiting fibroblast differentiation [49], has shown favorable results in Phase III clinical trials [50]. It has been demonstrated to significantly slow the decline in FVC in patients with IPF or PPF and is well tolerated [51], which represents a breakthrough for progressive fibrotic lung diseases and positions it as a promising therapy for CPFE as well. Beyond conventional oral agents, inhaled treprostinil has emerged as a promising novel therapy. On the basis of INCREASE trial (where CPFE/IPF represented 25.2% of participants), the global phase 3 TETON‐1 and TETON‐2 trials established its direct antifibrotic efficacy in IPF patients [52]. Over 52 weeks, inhaled treprostinil significantly attenuated median FVC decline compared with placebo (between‐group difference: 130.1 mL in TETON‐1 [*p* < 0.001]; 95.6 mL in TETON‐2 [*p* < 0.001]). Furthermore, inhaled treprostinil reduced the risk of clinical worsening by 33% in TETON‐1 (HR = 0.67, *p* = 0.003) and by 29% in TETON‐2 (HR = 0.71, *p* = 0.02) [53, 54].

#### 2.5.3 Bronchodilators and Inhaled Corticosteroids (ICS)

Regarding airway‐directed pharmacotherapy, evidence in CPFE is limited and recommendations are largely extrapolated from COPD and asthma guidelines [3]. Long‐acting bronchodilators, i.e., long‐acting muscarinic antagonists (LAMA) and long‐acting β_2_‐agonists (LABA), are the primary pharmacologic strategy for CPFE patients with coexisting airway obstruction consistent with COPD (post‐bronchodilator FEV_1_/FVC < 0.7) [55]. Methylxanthine derivatives such as theophylline may be considered as alternative or add‐on therapy when long‐acting bronchodilators are unavailable, insufficient, or intolerant, given their narrow therapeutic window and close monitoring requirement [56].

In contrast, ICS are mainly anti‐inflammatory and should generally not be used as monotherapy for airflow obstruction in CPFE. ICS may be considered as add‐on treatment within combination regimens (e.g., ICS/LABA or ICS/LABA/LAMA) for selected patients with asthma features, or those with recurrent/persistent exacerbations despite optimized long‐acting bronchodilator therapy [57]. Consistent with COPD‐oriented evidence, blood eosinophil count (EOS) may help identify patients more likely to benefit, with a greater expected benefit at blood EOS ≥ 300 cells/μL, and possible benefit at ≥ 100 cells/μL after dual bronchodilator treatment; these thresholds should be interpreted alongside asthma features, exacerbation history, and patient‐specific risks of ICS, particularly pneumonia [58].

#### 2.5.4 Symptomatic Support

Symptomatic and supportive care are key components of CPFE management. Oxygen therapy is fundamental, with most patients progressing from intermittent (exercise or nocturnal) to continuous oxygen use during the course. High‐flow or portable oxygen delivery devices enhance compliance and reduce the risk of hospitalization. Pulmonary rehabilitation as a comprehensive intervention (including respiratory muscle training, nutritional support, and psychological care) alleviates dyspnea, though exercise‐induced hypoxemia requires vigilance.

#### 2.5.5 Surgical Management

Volume reduction surgery or bronchial valve implantation is indicated only for early‐stage patients characterized by predominant upper‐lobe emphysema and minimal interstitial progression. Case reports suggest these procedures may improve exercise tolerance. Lung transplantation remains the only established intervention extending survival in end‐stage CPFE, with a 5‐year survival rate of approximately 50%–55%. Due to frequent coexisting pulmonary vascular disease and pleural adhesions, surgical complexity is high. Early referral to transplant centers is recommended, with continued antifibrotic therapy during the waiting period [59].

#### 2.5.6 Comorbidities Management

Pulmonary hypertension (PH) represents one of the most significant and frequent complications in patients with CPFE. For targeted vascular therapy, inhaled treprostinil has established robust evidence in patients with PH‐ILD [60]. In the INCREASE trial, 16 weeks of inhaled treprostinil therapy led to significant pulmonary vascular and clinical improvements, increasing 6‐min walk distance by a mean difference of 31.12 m (*p* < 0.001), reducing NT‐proBNP levels by 15% (vs. a 46% increase with placebo, *p* < 0.001), and decreasing the risk of clinical worsening by 39% (HR = 0.61, *p* = 0.04) [52]. Beyond these marked hemodynamic and clinical benefits in PH, inhaled treprostinil also possesses independent antifibrotic activity as discussed previously, highlighting its unique dual‐action potential in CPFE [52, 61]. Additionally, oral phosphodiesterase‐5 (PDE‐5) inhibitors such as sildenafil have shown potential in improving right ventricular function and quality of life in CPFE‐PH, although definitive long‐term survival benefits remain inconclusive [61].

Clinical cohorts and recent studies demonstrate that the prevalence of ischemic coronary artery disease (CAD) in CPFE ranges from 46.4% to 68% [62]. Mechanistically, the dual pulmonary insults of CPFE amplify hypoxic stress and pro‐inflammatory cytokine release, accelerating endothelial damage and atherosclerosis [47]. Integrated cardiopulmonary therapy is essential for CPFE patients with CAD to break the vicious cycle of hypoxia and inflammation [3]. Smoking cessation and long‐term oxygen therapy are foundational interventions [63]. Statins, antiplatelets, and selective β1‐blockers safely control CAD while alleviating systemic inflammation [64]. Timely antifibrotic therapy and inhaled bronchodilators can help manage CPFE without increasing cardiac risk [47]. PCI is the first‐choice revascularization strategy for severe stenosis [65]. Multidisciplinary surveillance lowers cardiovascular and all‐cause mortality, as CAD independently worsens CPFE prognosis [62].

CPFE patients exhibit elevated lung cancer (LC) risk with 1‐year survival rates below 50% [66]. CPFE‐UIP patients have a 2.69‐fold higher LC risk than IPF‐only cases [67]. CPFE‐LC patients demonstrate shorter median survival than COPD‐LC counterparts with higher mortality risk [59]. Even with complete resection and preserved lung function, the CPFE group exhibits a 16.7%–50% incidence of postoperative acute exacerbation (AE), significantly reducing long‐term survival [68]. A multidisciplinary team should evaluate surgical approaches and preoperative antifibrotic therapy. In addition, the risks of radiation pneumonitis should be noted in stereotactic radiotherapy.

Regarding AE‐CPFE, clinical presentations can be differentiated into “AE‐COPD‐type” and “AE‐IPF‐type” [69]. The former is primarily driven by infection or airway spasm and is treated with systemic corticosteroids combined with broad‐spectrum antibiotics and enhanced bronchodilation. The latter may involve diffuse alveolar damage, for which high‐dose steroid pulse therapy followed by rapid tapering is recommended. Adjunctive treatments such as cyclophosphamide or tocilizumab may be considered, although supporting evidence is limited. Despite aggressive management, mortality remains high among patients experiencing AE‐IPF‐type events [70].

In terms of SARD‐CPFE, treatment should address not only pulmonary manifestations but also the underlying SARD. Therefore, patients with SSc, RA, PM/DM, or AAV may benefit from disease‐specific immunomodulatory regimens. Therapeutic options include glucocorticoids, mycophenolate mofetil (MMF), azathioprine, cyclophosphamide, tacrolimus, and biologics such as rituximab, depending on SARD subtype and systemic involvement. Early identification of autoantibody profiles assists in phenotyping the underlying SARD, predicting disease trajectory, and guiding personalized immunosuppressive strategies [71].

For patients with iCPFE or those with telomere‐related mutations, surfactant‐associated gene defects, or abnormalities in TGF‐β signaling, management should include screening for systemic features of short‐telomere syndrome and family history assessment. Supportive care remains central, but genetic counseling may be offered in selected cases [72]. Given that telomere dysfunction is associated with heightened sensitivity to cytotoxic immunosuppressants, caution is warranted when considering cyclophosphamide or azathioprine in mutation‐positive patients, and MMF or targeted biologics may be preferred [73].

### 2.6 Prognosis of CPFE

Variations in sample size, disease severity, and follow‐up duration across studies contribute to heterogeneity in reported median survival time. The main causes of death in CPFE patients are PH, LC, and AE [3]. PH is one of the most common complications of CPFE and reduces the 5‐year survival rate from 75% to 25% [1, 25]. CPFE‐LC patients had a median survival of 19.5 months, much shorter than in non‐CPFE patients (53.1 months) [74]. One study reported that 1‐, 3‐, and 5‐year survival rates in CPFE‐LC patients were 52%, 40%, and 37%, respectively [75]. “AE‐IPF‐type” has a worse prognosis than “AE‐COPD‐type” [69]. Moreover, CAD is not only a critical extrapulmonary complication but also a poor prognostic factor in CPFE [3], accounting for 11.3% of all deaths in CPFE cohorts [63]. Concurrent CAD further exacerbates the cardiopulmonary burden and triggers AE or sudden death [64]. Therefore, routine screening and comprehensive management of CAD are strongly recommended in CPFE patients [76].

The progression of emphysema or fibrosis has different effects on CPFE outcomes [77]. In terms of emphysema progression, when emphysema severity was ≥ 10%, the annual decline rates of FVC and DLCO were greater in patients with CPFE than in those with IPF and were associated with a worse prognosis [78]. However, in an analysis of IPF patients, there was no association between the presence or extent of emphysema and survival after adjustment for baseline disease severity [79]. Intriguingly, a greater extent of emphysema was associated with a better prognosis, suggesting a potential “protective effect” for CLE [80, 81]. In contrast, fibrosis progression may represent a poor prognosis. For instance, a quantitative study identified fibrosis extent as an independent risk factor: The group with ≥ 10% fibrosis exhibited the poorest overall survival and highest AE rate, whereas patients with < 5% fibrosis displayed relatively benign disease behavior regarding overall survival and lung function preservation [82]. A cohort study reported that patients with ILD extent ≥ 30% had shorter survival than those with < 30% [62]. On CT imaging, a honeycombing extent > 50% serves as an independent predictor of adverse outcomes [83], as does the fibrosis‐weighted CT index [84]. Prolonged (> 12 months) antifibrotic therapy is associated with improved prognosis [85]. Additionally, a pulmonary artery‐to‐aorta diameter ratio (PA/AA) > 1, as a surrogate marker for PH, is associated with reduced survival [86]. Therefore, it is important to delineate abnormal imaging findings in CPFE patients to predict disease prognosis.

Several clinical features and indicators are associated with the prognosis of CPFE. Digital clubbing and mediastinal lymphadenopathy predict poor prognosis [77, 87]. Severe impairment of gas exchange capacity is a prominent feature of CPFE, and patients with decreased DLCO have a significantly shorter survival time [88]. In a cohort study, patients with DLCO (%pred) ≥ 39% had longer survival than those with DLCO (%pred) < 39% [62]. Other studies indicated that FVC (%pred) < 50%, FEV_1_ (%pred)/FVC (%pred) > 1.2, and lower VC (%pred) are associated with poor prognosis [23, 87, 89, 90]. Elevated cytokeratin 19 fragment (CYFRA21‐1) and CA125 levels are associated with mortality in CPFE [85]. Elevated KL‐6, decreased SP‐D, and reduced arterial oxygen partial pressure (PaO_2_) are also important adverse prognostic factors [91]. Systolic pulmonary artery pressure (sPAP) ≥ 75 mmHg, right ventricular diameter (RVD), C‐reactive protein (CRP), and globulin are also associated with prognosis [24, 88]. In addition, both elevated neutrophil‐to‐lymphocyte ratio (NLR) and elevated carcinoembryonic antigen (CEA) level are associated with increased all‐cause mortality risk in patients with CPFE‐LC [75].

### 2.7 Future Perspectives on the Evaluation and Management of CPFE With AI

Previous research suggests that the future management of CPFE is moving toward precision‐based intervention, which will depend on integrating multidimensional data from the clinical, imaging, physiologic, and molecular levels [47]. At present, however, CPFE‐specific AI research remains limited. Accordingly, this section is intentionally forward‐looking and draws heavily on evidence from better‐studied diseases such as IPF, COPD, and other ILDs. Given the limitations of traditional methods for longitudinally quantifying and monitoring CPFE, AI may improve disease identification, risk stratification, and personalized assessment. Exploring AI‐based evaluation and management in CPFE is therefore of considerable interest (shown in Figure 4).

**FIGURE 4:**
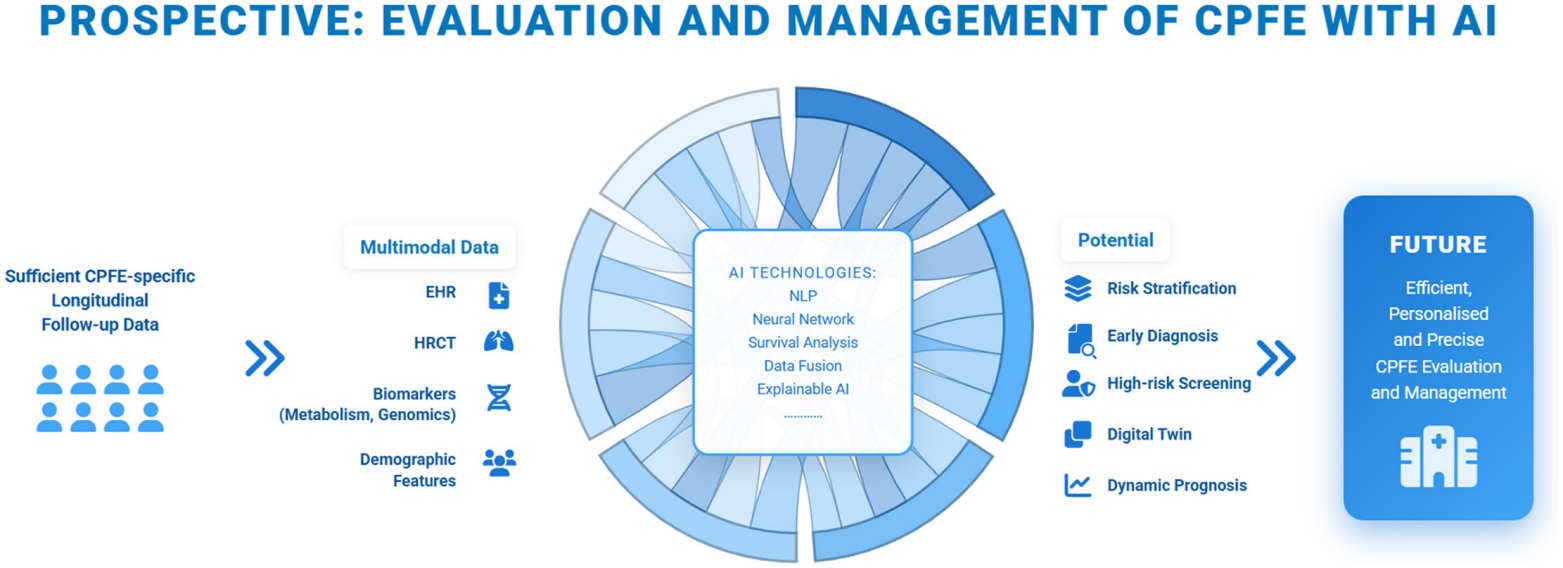
The prospects of evaluation and management of CPFE with AI. Sufficient CPFE‐specific longitudinal follow‐up data serve as the foundation for model development. Multimodal data—including electronic health records, high‐resolution computed tomography, metabolic and genomic biomarkers, and demographic features—are integrated to enable a range of AI‐driven clinical applications. These applications include risk stratification, early diagnosis, high‐risk screening, digital‐twin modeling, and dynamic prognosis prediction. Together, these components aim to achieve efficient, personalized, and precise CPFE evaluation and management, supported by tools such as natural language processing, neural networks, survival analysis, data fusion, and explainable AI. EHR = electronic health records; NLP = natural language processing.

#### 2.7.1 AI‐Assisted Evaluation

The main value of AI in CPFE assessment is likely to lie in three areas: high‐precision quantitative analysis, accurate structural classification, and multimodal information integration. HRCT‐based AI has already demonstrated strong capability in identifying complex pulmonary structural alterations [92, 93, 94, 95, 96]. Qiu et al. employed a multichannel RadCT‐CNNViT ensemble network to achieve high‐precision classification of pulmonary nodular disease and LC [93], demonstrating the potential of deep learning in capturing complex chest imaging features. For CPFE, AI can further advance automated quantitative processing of HRCT, including precise segmentation of fibrosis and emphysema, identification of mixed structures, and radiomics extraction of subtle imaging textures. This could reduce the subjectivity of image interpretation, improve consistency in phenotype characterization, and contribute to a standardized CPFE imaging evaluation system. Additionally, AI can integrate multimodal data such as imaging, pulmonary function, blood gas analysis, biomarkers, and medical history to identify potential correlations among them. Multiple studies have demonstrated the superior performance of such multimodal models in the differential diagnosis and prediction of disease progression in complex ILDs [97, 98, 99]. This approach may help establish a more biology‐driven classification system for CPFE, revealing the risk patterns and evolutionary trajectories of different subtypes, thereby enabling more precise assessments of disease severity and prognosis.

Because CPFE is a dynamic disease, static information at diagnosis is often insufficient for accurately predicting progression. Time‐series deep learning models, such as RNNs, LSTMs, and Transformers, together with dynamic survival analysis techniques based on longitudinal follow‐up data, can continuously update risk estimates throughout follow‐up. This may better capture the rate of lung function decline, trends in radiographic deterioration, and the risk of AE. Relevant studies have already shown the feasibility of such models in ILD research [100, 101, 102, 103, 104], providing a transferable technical pathway for dynamic prognostic prediction in CPFE.

#### 2.7.2 AI‐Assisted Management

The management of CPFE is highly complex because it often coexists with parenchymal destruction, fibrotic remodeling, emphysema, PH, and malignant tumors [47]. Traditional management relies on periodic imaging, pulmonary function testing, and clinician judgment, which is both time‐consuming and resource‐intensive. AI‐driven management tools may help optimize this process through precise stratification, dynamic monitoring, and personalized interventions.

First, AI may serve as a clinical decision‐support tool by integrating multimodal data to help refine treatment selection. For example, it may be able to predict responsiveness to antifibrotic therapies or identify early risks of PH, thereby supporting more proactive monitoring and timely intervention. It may also improve multidisciplinary collaboration and support a more rational allocation of specialist resources. Among these approaches, natural language processing (NLP) is particularly relevant for CPFE management. NLP applied to electronic health records (EHRs) or electronic medical records (EMRs) can automatically extract key medical histories, symptom progression, physiologic indicators, and treatment responses from unstructured text. This capability is especially critical for CPFE, which exhibits diverse etiologies and heterogeneous progression, since many important indicators, such as smoking history, rheumatologic/immunologic background, and PH risk factors, are often scattered throughout lengthy medical records. Existing research demonstrates the strong performance of NLP in respiratory disease management [105, 106, 107, 108, 109]. For instance, Liang et al. [108] utilized NLP to accurately extract FVC values from EHRs, increasing the availability of longitudinal records and proving its feasibility for automated follow‐up and trend analysis.

An emerging frontier is the development of digital‐twin models [110, 111]. In this approach, a virtual model is created for each CPFE patient, integrating multimodal data and allowing different therapeutic strategies to be tested in silico. Such models may provide a more structured way to evaluate the potential benefits and risks of various interventions. In particular, when clinicians encounter uncommon CPFE phenotypes or atypical lesions, digital twins may offer a framework for comparing alternative treatment options and supporting more personalized and dynamically optimized management.

Although AI presents various opportunities for precise assessment and personalized management of CPFE, it still faces several critical limitations. The limited scale of CPFE‐specific data, insufficient longitudinal follow‐up, and lack of multimodal information compromise the reliability of model training and external validation. Furthermore, variations across centers in imaging acquisition, pulmonary function measurement, and clinical information recording restrict the models' generalization capabilities. Many cutting‐edge technologies, such as longitudinal dynamic modeling, NLP, and digital twins, remain in exploratory phases without established application pathways. With the development of larger‐scale data resources, improvements in algorithmic performance, and advancement of cross‐center collaboration, AI is poised to become a vital pillar for precise diagnosis, risk assessment, and dynamic management of CPFE in the future.

## 3 Conclusion

CPFE is a highly heterogeneous disease characterized by significant variations in biological mechanisms, imaging features, and clinical manifestations. Traditional diagnostic approaches, based solely on imaging or pulmonary function tests, fail to fully capture its pathological complexity. Consequently, future research should prioritize precision classification grounded in etiologic mechanisms. This underlying heterogeneity not only influences disease natural history but also determines patient variability in response to antifibrotic drugs or immunomodulatory therapies. By elucidating the molecular pathways and cellular biology underlying the disease development, we may transform CPFE classification from phenotype‐based definitions to mechanism‐driven categorization, providing clearer directions for targeted personalized therapy.

An AI model integrating HRCT‐based quantitative imaging features, longitudinal functional follow‐up data, and multiple sets of biomarkers can capture disease progression patterns across the entire disease course, identify high‐risk populations, and provide quantitative evidence for clinical decision‐making. With the accumulation of multicenter data resources and the maturation of AI algorithms, CPFE is expected to move from a “vague syndrome” to an era of precision pulmonology characterized by “quantifiable, interpretable, and predictable” disease management.

## Author Contributions

F.L. and Q.D. conceived this review. Q.D., C.C., Q.Z., and Z.W. wrote the manuscript. F.L. and J.S. reviewed and edited the manuscript. All authors contributed to figure preparation and manuscript revision and reviewed and approved the final manuscript.

## Funding

This study was funded by Shanghai Municipal Health Commission (No. 20244Z0019), Physician‐scientist project of Shanghai Jiao Tong University School of Medicine (No. 20240820), the Student Innovation Training Program of Shanghai Jiao Tong University School of Medicine (No. 20260621X), Shanghai Municipal Medical Outstanding Young Medical Talent Training Program, and Shanghai Jiao Tong University Cultivation Platform for Future Cross‐Disciplinary Innovative Talents. The funder had no role in the design, data collection, data analysis, or reporting of this study.

## Ethics Statement

An ethics statement is not applicable because this study is based exclusively on published literature.

## Conflicts of Interest

The authors declare no conflicts of interest.

## Data Availability

No datasets were generated or analyzed during the current study.
